# Missing lateral relationships in top-level concepts of an ontology

**DOI:** 10.1186/s12911-020-01319-3

**Published:** 2020-12-15

**Authors:** Ling Zheng, Yan Chen, Hua Min, P. Lloyd Hildebrand, Hao Liu, Michael Halper, James Geller, Sherri de Coronado, Yehoshua Perl

**Affiliations:** 1 Computer Science and Software Engineering Department, Monmouth University, West Long Branch, NJ 07764 USA; 2 CIS Department, Borough of Manhattan Community College, CUNY, New York, NY 10007 USA; 3 Department of Health Administration and Policy, George Mason University, Fairfax, VA 22030 USA; 4 Union Square Eye Care, New York, NY 10003 USA; 5 Department of Computer Science, New Jersey Institute of Technology, Newark, NJ 07102 USA; 6 Department of Informatics, New Jersey Institute of Technology, Newark, NJ 07102 USA; 7 National Cancer Institute, Center for Biomedical Informatics and Information Technology, National Institutes of Health, Rockville, MD 20850 USA

**Keywords:** Ontology quality assurance, Ontology modeling, Missing relationship error, Omission error, Error concentration, Abstraction network, Taxonomy, National Cancer Institute thesaurus (NCIt), SNOMED CT

## Abstract

**Background:**

Ontologies house various kinds of domain knowledge in formal structures, primarily in the form of concepts and the associative relationships between them. Ontologies have become integral components of many health information processing environments. Hence, quality assurance of the conceptual content of any ontology is critical. Relationships are foundational to the definition of concepts. *Missing relationship errors* (i.e., unintended omissions of important definitional relationships) can have a deleterious effect on the quality of an ontology. An abstraction network is a structure that overlays an ontology and provides an alternate, summarization view of its contents. One kind of abstraction network is called an *area taxonomy*, and a variation of it is called a *subtaxonomy*. A methodology based on these taxonomies for more readily finding missing relationship errors is explored.

**Methods:**

The *area taxonomy* and the *subtaxonomy* are deployed to help reveal concepts that have a high likelihood of exhibiting missing relationship errors. A specific top-level grouping unit found within the area taxonomy and subtaxonomy, when deemed to be anomalous, is used as an indicator that missing relationship errors are likely to be found among certain concepts. Two hypotheses pertaining to the effectiveness of our Quality Assurance approach are studied.

**Results:**

Our Quality Assurance methodology was applied to the *Biological Process* hierarchy of the National Cancer Institute thesaurus (NCIt) and SNOMED CT’s *Eye/vision finding* subhierarchy within its *Clinical finding* hierarchy. Many missing relationship errors were discovered and confirmed in our analysis. For both test-bed hierarchies, our Quality Assurance methodology yielded a statistically significantly higher number of concepts with missing relationship errors in comparison to a control sample of concepts. Two hypotheses are confirmed by these findings.

**Conclusions:**

Quality assurance is a critical part of an ontology’s lifecycle, and automated or semi-automated tools for supporting this process are invaluable. We introduced a Quality Assurance methodology targeted at missing relationship errors. Its successful application to the NCIt’s *Biological Process* hierarchy and SNOMED CT’s *Eye/vision finding* subhierarchy indicates that it can be a useful addition to the arsenal of tools available to ontology maintenance personnel.

## Background

Ontologies provide foundational terminological support for various systems and processes in the biomedical field, including electronic health records (EHRs) [1], decision-support systems [2], and data integration [3]. Ontologies are typically composed of a large collection of *concepts* that are interlinked by various *lateral relationships* (*relationships*, in short) expressing associative knowledge. As an example, in the National Cancer Institute thesaurus (NCIt), the concept *Breast Neoplasm* is connected to the concept *Breast* via the relationship *Disease Has Associated Anatomic Site*, explicitly denoting the anatomic site where breast neoplasm is found. Given ontologies’ growing use, assuring the quality of ontological content is critical. Examples of content problems include incorrectly defined concepts, misclassified concepts, and incorrect synonymy. All the preceding are errors of commission. In this work, we are focusing on quality assurance (QA) pertaining to a specific kind of error of omission, namely, *missing relationship errors*, i.e., omissions of critical relationships from concept definitions. We are interested in mechanisms for identifying sets of concepts that are highly likely to be in this state of under-definition. While it is true that some consider an error of omission as being less severe than an error of commission, missing relationship errors can nonetheless have a deleterious effect on the quality of the ontology, particularly when they appear in large numbers. Moreover, as relationships affect the functioning of classifiers employed in ontology management, omitted relationships can lead to the incorrect placement of concepts (i.e., incorrect parentage) in the ontology hierarchy [4].

In previous work, we have developed a number of abstraction networks—compact summarization structures for ontologies—and have shown them to be useful in support of ontology QA [5]. In particular, the alternative view of an ontology offered by an abstraction network supports the identification of sets of concepts with high likelihood of errors. For example, a number of abstraction networks, particularly those that we refer to as *taxonomies* [6–8], have been developed for very large ontologies with hundreds of thousands of concepts, e.g., National Cancer Institute thesaurus (NCIt) [9], the Gene Ontology (GO) [10], SNOMED CT [11], Chemical Entities of Biological Interest (ChEBI) [12], Uberon [13], and National Drug File-Reference Terminology (NDF-RT) [14]. They have also been used on some relatively small ontologies with at most thousand concepts, such as the Ontology of Clinical Research (OCRe) [15], the Sleep Domain Ontology (SDO) [16], the Ontology for Drug Discovery Investigations (DDI) [17], and the Cancer Chemoprevention Ontology (CanCo) [18]. The Ontology Abstraction Framework (OAF) tool [19] enables the automatic derivation of taxonomies for many BioPortal hosted ontologies [20].

In this paper, we deploy a type of abstraction network called an *area taxonomy* and one of its variations called a *subtaxonomy* in our efforts to uncover missing relationship errors. Both abstraction networks serve to group together concepts having similar relationship configurations. In this way, they each make it easier to discern concepts that collectively exhibit this kind of similarity. In both cases, the focus of our efforts is on high-level concept groupings, called *top areas*. These groupings typically comprise concepts with minimal sets of relationships for the particular hierarchy or subhierarchy. From a modeling perspective, a top area contains the root of the hierarchy and in addition is expected to include other general concepts. The number of general concepts is expected to be a small percentage of the overall hierarchy. If, however, the top area has a large number of concepts, then this is a natural place to search for missing relationship errors. Moreover, we consider the hierarchical depth of a top area as a factor in our approach. The deeper down a concept is in the top-area hierarchy, the more suspicious it is.

We note that the area taxonomy and the subtaxonomy are not by themselves providing QA methodologies, but instead are serving as frameworks for describing our QA approaches. One such methodology (using top-areas) is presented in this paper, while other such QA methodologies using alternate sets of candidate concepts with high likelihoods of errors have previously been employed (see, e.g., [21, 22]).

Our methodology is demonstrated using two test-beds. The first is the NCIt’s *Biological Process* hierarchy (15.02d release), having a total of 1145 concepts. The area taxonomy analysis is applied to this complete hierarchy. The second is the “*Eye/vision finding*” subhierarchy of the *Clinical finding* hierarchy of SNOMED CT. In the January 2018 release used in the study, the *Clinical finding* hierarchy has 111,081 concepts; its “*Eye/vision finding*” subhierarchy has 5812 concepts. The subtaxonomy analysis is done on this subhierarchy. Both test-beds were chosen because their top areas are proportionally large in size. The *Biological Process* top area contains about 45% of the hierarchy’s concepts. The *Eye/vision finding* top area has 22% of the subhierarchy’s concepts.

It is interesting to point out that the top area of the NCIt *Biological Process* hierarchy was not always that large. In the year 2004 [6], only 47 concepts out of its 589 concepts (8%) were in the top area. By the time of the 15.02d release, the *Biological Process* hierarchy had a total of 1145 concepts, of which 513 (45%) were in the top area. That is, while the *Biological Process* hierarchy grew about two-fold, the top area grew about 11-fold. When we see such disproportionate growth of the top area, it can be interpreted as an anomaly alerting us to the possibility of widespread missing relationship errors. Indeed, our findings in the context of the *Biological Process* hierarchy include many such errors, confirmed by the curators of the NCIt, as described herein.

Hypotheses pertaining to the efficacy of the methodology are proposed and the confirmed results analyzed with respect to these. The implications of correcting missing relationship errors at the upper reaches of hierarchies and subhierarchies are explored. The application of our methodology to other NCIt and SNOMED CT hierarchies is discussed. A preliminary description of the NCIt results appeared previously [23]; however, that presentation was different and did not use the area taxonomy framework.

### Ontology concepts and lateral relationships

**Fig. 1:**
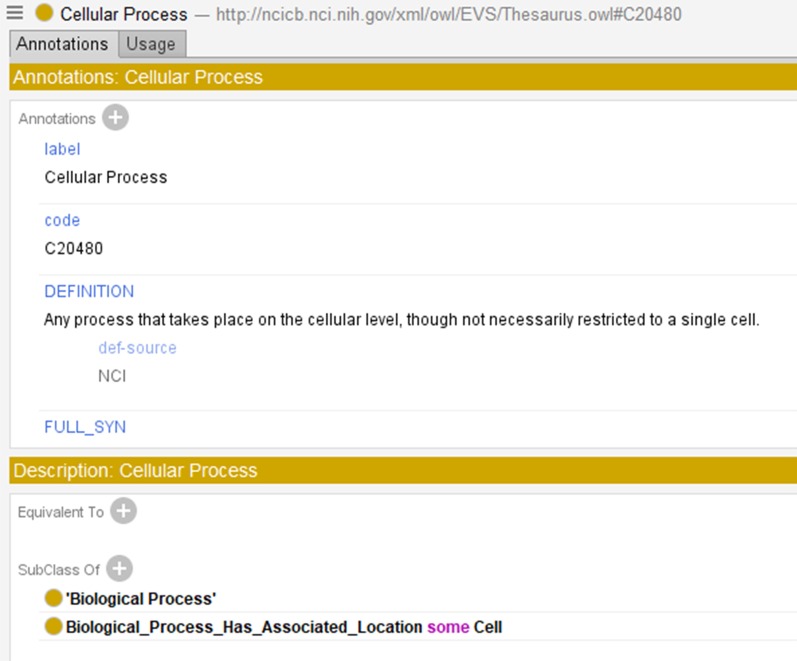
Concept *Cellular Process* from NCIt shown in Protégé, including the subclass (IS-A) relationship to *Biological Process*, and the relationship (role) *Biological Process Has Associated Location* to *Cell*

The building blocks of an ontology are its concepts. And concepts connect with other concepts through the hierarchical IS-A (subsumption) relationships to form the ontology’s overall hierarchy. Some ontologies, like NCIt and SNOMED CT, have multiple, independent hierarchies with respective top (root) concepts. Lateral relationships are non-hierarchical relationships that also connect concepts—source concepts to target concepts—and serve as foundational definitional elements for source concepts. A lateral relationship between a pair of concepts is expressed by a triple of the form (*c*_1_, *c*_2_, *r*), where *c*_1_ is the source concept, *c*_2_ is the target concept, and *r* is the relationship name. Such a triple is called a *role* in the context of the NCIt, an *attribute relationship* in SNOMED CT, and an *object property* in OWL ontologies. Figure 1 shows the axiomatic description of the concept *Cellular Process* from the NCIt using the Protégé interface [24], including the relationship (role) specification for *Biological Process Has Associated Location*.

### NCIt and SNOMED CT

The NCIt is an ontology mainly focused on cancer-related concepts. However, as the need for non-cancer applications has increased, the NCIt has been including an increasing number of non-cancer concepts and has become a widely recognized biomedical standard used by a broad variety of public and private organizations, both nationally and internationally.

NCIt is developed with Protégé 3.5 (Protégé OWL) and is modeled using description logic (DL) [25, 26]. We used the OWL version 15.02d of the NCIt in this work. This version contains 108,376 active concepts organized into 19 IS-A hierarchies, including, e.g., *Disease, Disorder or Finding*; *Anatomic Structure, System, or Substance*; *Drug, Food, Chemical or Biomedical Material*; *Biological Process*; and *Gene*. Each concept belongs to exactly one hierarchy, though there can be multiple inheritance within a given hierarchy.

For each NCIt hierarchy, there is a list of prescribed relationships that can be associated with its concepts. In this study, we focused on the *Biological Process* (BP) hierarchy, containing 1145 concepts with seven possible associated relationships (whose full names and abbreviated names are given in Table 1).

**Table 1:** Relationships in NCIt’s *Biological Process* hierarchy and their abbreviations

Relationship	Abbreviated name
*Biological Process Has Associated Location*	*Location*
*Biological Process Has Initiator Chemical Or Drug*	*Initiator Chemical or Drug*
*Biological Process Has Initiator Process*	*Initiator BP*
*Biological Process Has Result Anatomy*	*Resulting Anatomy*
*Biological Process Has Result Biological Process*	*Resulting BP*
*Biological Process Has Result Chemical Or Drug*	*Resulting Chemical or Drug*
*Biological Process Is Part Of Process*	*Part of Process*

SNOMED CT is a widely used international standard ontology. The release we worked on is the January 2018 International Edition including 341,105 concepts connected by 511,767 IS-A relationships and 1,527,383‬ lateral relationships. SNOMED CT’s concepts are organized into 19 major hierarchies (e.g., *Clinical finding* and *Procedure*). The *Clinical finding* hierarchy is the largest hierarchy in SNOMED CT with 111,081 concepts. This hierarchy has a list of 17 prescribed relationship types for the definition of its concepts. In this paper, we focus on the *Eye/vision finding* subhierarchy of *Clinical finding*. This subhierarchy has 5812 concepts defined in term of 15 possible relationship types.

### Area taxonomy

An abstraction network of an ontology is a compact network designed to summarize its structure and semantics. The summarization is in the form of a smaller network of nodes representing units of concepts identified to be structurally and semantically similar according to certain criteria. In previous work, we have demonstrated that various kinds of abstraction networks can be utilized to support ontology QA. One kind of abstraction network is the *area taxonomy* [5], whose constituent network is composed of nodes called *areas* and links denoted *child-of*.

**Fig. 2:**
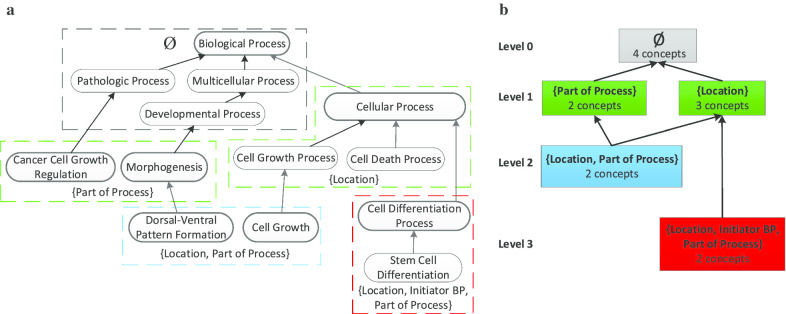
**a** Excerpt of 13 concepts from the NCIt’s *Biological Process* hierarchy. Upward arrows represent IS-A relationships. Concepts with the same set of relationships are enclosed in a common, colored area. E.g., *Cancer Cell Growth Regulation* and *Morphogenesis* have one relationship *Part of Process*. Areas with the same number of relationships have the same color. E.g., the area {*Location*} and the area {*Part of Process*} are green. Area roots, e.g., *Cellular Process*, have bold outlines. **b** Area taxonomy for **a**, composed of five areas. Areas are represented by colored boxes labeled with their sets of relationships and numbers of concepts. They are organized in color-coded levels, according to number of relationships. The three concepts having the *Location* relationship are now represented by an area box named {*Location*}. *Child-of* links between areas are bold arrows; e.g., {*Location*, *Part of Process*} on Level 2 and {*Location*, *Initiator BP*, *Part of Process*} on Level 3 are *child-of* area {*Location*}

**Fig. 3:**
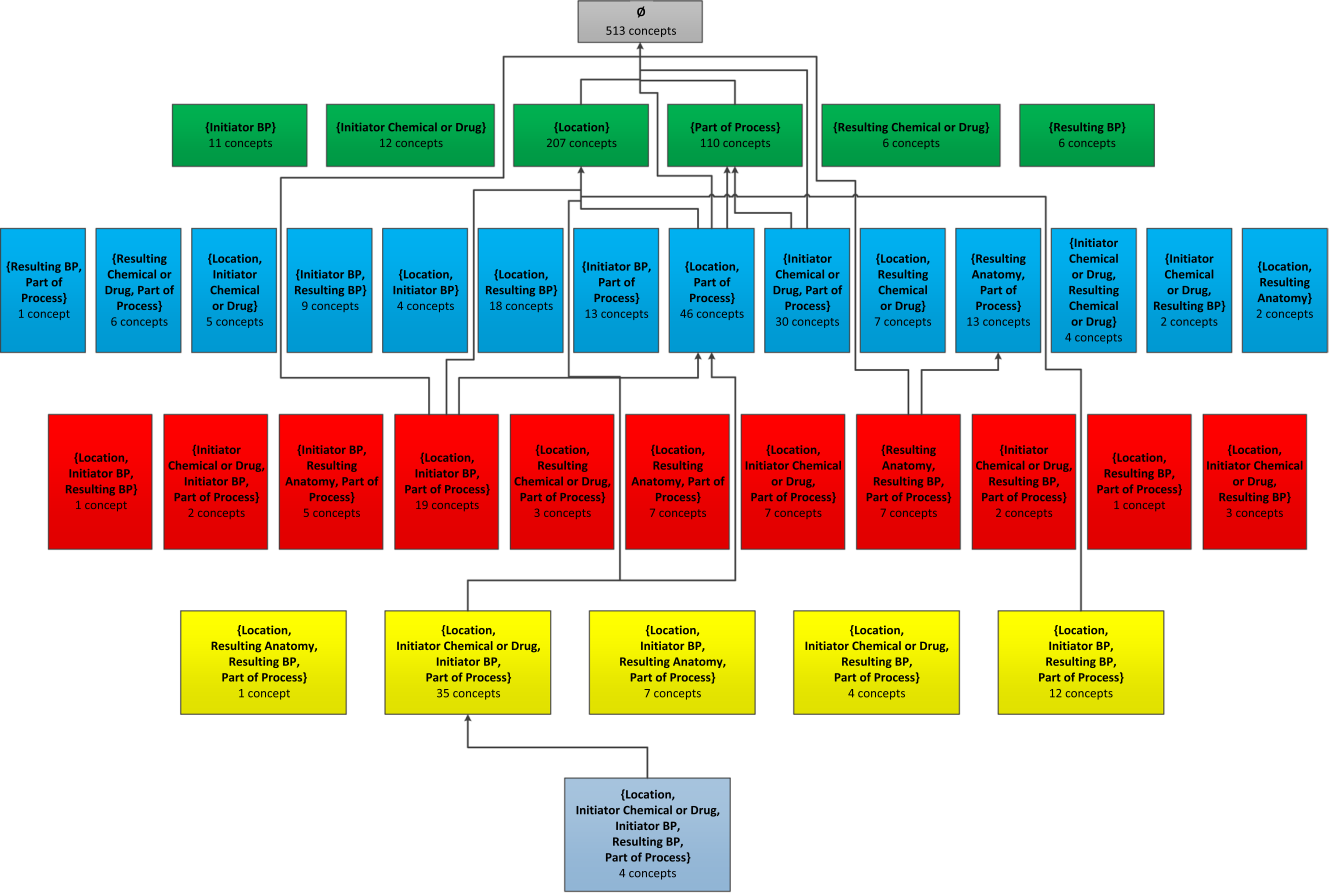
Complete area taxonomy for the NCIt’s *Biological Process* hierarchy. Most *child-of’*s have been omitted to avoid overload. Note how the importance of the relationship *Location* is reflected in the area taxonomy. Area {*Location*} has 207 concepts, and *Location* appears in 20 of 37 area names

An area (node) denotes the non-empty set of all concepts having exactly the same group of defined lateral relationships. For example, in NCIt’s *Biological Process* (BP) hierarchy, certain concepts (e.g., *Protein Expression*) have exactly the three relationships *Location*, *Initiator BP*, and *Part of Process* (and no others). Hence, there is an area named {*Location*, *Initiator BP*, *Part of Process*} containing those concepts. The top area in this context contains all concepts having no lateral relationships at all. Each concept can reside in only one area; thus, areas are disjoint. A root of an area is a concept having no parent concepts in its area. An area has one or more roots. *Child-of* hierarchical links connecting areas are derived based on the underlying concept hierarchy in the ontology. Specifically, an area *A* is *child-of* another area *B* if a root in *A* has a parent in *B*. Figure 2 illustrates the derivation of the area taxonomy for an excerpt of 13 concepts from the BP hierarchy. Figure 3 shows BP’s complete area taxonomy. Note that in Fig. 2b there is a *child-of* from Level 3 to Level 1, due to the addition of two relationships at the two concepts. Similarly, many *child-of* relationships in Fig. 3 are between non-adjacent levels.

### Subtaxonomy

Although an area taxonomy of a hierarchy is more compact than the hierarchy itself, the complete area taxonomy for the whole *Clinical finding* hierarchy of SNOMED CT contains 524 areas due to its large number of relationship types. To obtain more manageable summarizations of such a large hierarchy, we can use a divide and conquer approach and apply the area taxonomy abstraction technique on a chosen subhierarchy [27] to obtain a *subtaxonomy*.

**Fig. 4:**
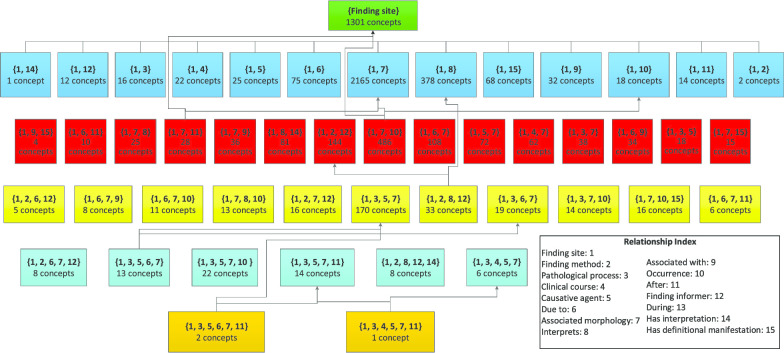
An excerpt of the subtaxonomy for the *Eye/vision finding* subhierarchy in SNOMED CT, presenting 48 areas out of 97 areas in the complete subtaxonomy

The derivation of a subtaxonomy is the same as for an area taxonomy. The root *c* of the subhierarchy is the uppermost concept considered*.* The root area in the subtaxonomy consists of the concept *c* and all its descendants having the exact same relationships as *c*. For example, the subtaxonomy for the subhierarchy rooted at *Eye/vision finding* (used as a test-bed in this paper) has a top area with 1301 concepts, all having the one relationship *Finding site*. Overall, its 5812 concepts are divided into 97 areas. An excerpt of the subtaxonomy for *Eye/vision finding* is shown in Fig. 4.

## Methods

### Area taxonomy-based technique to identify concepts more prone to miss relationships

As noted, each NCIt and SNOMED CT hierarchy has a defined group of relationships that can be used in modeling the hierarchy’s concepts. Table 1 lists the seven relationships available in the NCIt *Biological Process* (BP) hierarchy. For example, the BP concept *Protein Expression* has the three relationships *Location, Initiator BP* and *Part of Process.*

Curators of ontologies rarely have the resources for QA of a complete ontology. However, they can be aided by tools that propose suspicious concepts that require their attention. Such tools flag concepts with specific characteristics that indicate a higher error probability. Examples of such characteristics are overlapping concepts [28, 29], concepts with many relationships [30] and concepts in small subgroups within the area taxonomy [6, 21, 31]. For details of those characteristics, see the relevant references. By comparing many area taxonomies and subtaxonomies, it was realized that "residing in the top area of a taxonomy" is also likely to be one such characteristic, because this does not commonly happen for many concepts [5]. In other words, when the top area of an area taxonomy (or of a subtaxonomy) is large, relative to the whole taxonomy, this indicates an anomaly, because a high percentage of concepts in the hierarchy (or subhierarchy) have no (or very few) relationships. This makes it highly likely that they were "under-defined" in the first place.

The NCIt *Biological Process* hierarchy demonstrates such a situation. When concepts *legitimately* do not have any relationships, they typically capture general classes for which no relationships need to be modeled, e.g., *Pathologic Process* and *Reproductive Process*. Typically, such concepts reside immediately under the hierarchy’s root (*Biological Process* for these two concepts) or are close to it. However, most meaningful and useful concepts are expected to have relationships. We propose that a top area of an area taxonomy (or a subtaxonomy) with relatively many concepts is an indication that many of those concepts are missing lateral relationships. This idea can be formalized as follows.

#### Hypothesis 1

If a large percentage of concepts of a hierarchy (or subhierarchy) appear in the top area of an area taxonomy (or subtaxonomy), then the percentage of concepts in this top area that are missing relationships is statistically significantly higher than the percentage of such concepts in other areas.

We conducted two studies to assess this hypothesis. In the first study, focused on the NCIt’s *Biological Process* hierarchy, the QA analysis was performed for all its 513 top-area concepts (44.8% of the overall hierarchy). As a control sample, we used 100 concepts randomly selected from all areas except for the top area. Taking into consideration previous research on this hierarchy [6], we also excluded another anomaly called "small partial-areas," so as not to bias this study.

The study was carried out manually by one of the authors (YC), who has medical and ontological training and extensive experience in ontology QA. We are not familiar with any published automatic method to determine missing relationships. A manual review by a domain expert is required, since human understanding and domain expertise are needed for such judgements. However, the detection of sets of concepts with high likelihood of errors can be performed algorithmically. The missing relationship errors found by YC were submitted for a secondary review to another author (SdC), who is in charge of the NCIt team.

A second QA study was performed on the SNOMED CT’s *Eye/vision finding* subhierarchy. Co-author (HM) with training in medicine and biomedical ontologies and extensive experience in QA of ontologies, reviewed a random sample of 96 top area concepts and 96 concepts from other areas. The resulting error report included concepts with missing relationship errors and corresponding correction suggestions. The American Academy of Ophthalmology (AAO) had previously initiated a project for enriching SNOMED coverage of ophthalmology, which consisted at that time of about 2000 concepts. Co-author (PLH), an ophthalmologist who was the Head of the IT committee of the AAO, spearheaded this project. During 2001 to 2008, the AAO team contributed 9510 unique or preferred terms, and 5223 synonyms for ophthalmology concepts which were inserted into SNOMED [32] by Dr. Spackman, the SNOMED CT chief ontologist at the time. Thus, we have recruited PLH to be the second authoritative reviewer for the error report. He reviewed and confirmed HM’s error report but also found more missing relationships in the sample. The statistical analysis to evaluate Hypothesis 1 was preformed based on the combined results of these two-step reviews.

### A complexity measure to prioritize top area concepts more likely to miss relationship

In some area taxonomies (or subtaxonomy), even the top area by itself is too large to make a QA review by a human expert a practical possibility. As a case in point, the taxonomy of the *Disease, Disorder, or Finding* hierarchy of NCIt contains in its top area 14,347 concepts (out of 25,360). Similarly, the top area in the *Eye/vision finding* subhierarchy of SNOMED CT has 1301 concepts. In such a case, the challenge is to narrow down the QA effort to a more promising subset of the top area. For this purpose we employ another theme called “complexly modeled concepts.”

While a concept with no relationships is likely to be under-modeled, a concept with many relationships is "complex" and therefore more likely to be modeled incorrectly. A concept of higher complexity is more likely to contain an error than a simpler concept and one way to measure the complexity of a concept is by its number of relationship types.

**Fig. 5:**
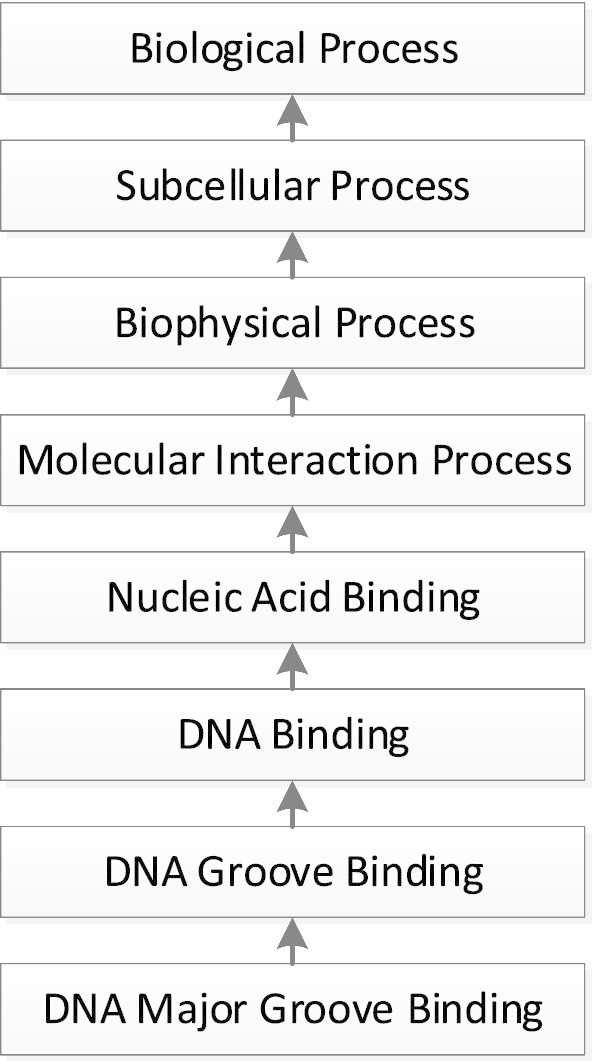
Path of seven IS-As to the root in the NCIt *Biological Process* hierarchy

A concept with six relationship types is likely to be more complex than a concept with, say, one or two relationship types, and thus there is a higher likelihood of introducing a modeling error for the former [30]. However, this method of measuring complexity is not applicable to the top area, where concepts have no relationships. (For a subhierarchy, all concepts in the top area have the same number of relationship types, which also does not lend itself to distinguish between them.) To overcome this issue, we introduce a novel characteristic that captures concept complexity. Consider the hierarchical distance of concepts of the top area to the root concept of the top area. Figure 5 shows an example of a hierarchical path in the top area of the NCIt *Biological Process* hierarchy.

In this example, the concept *DNA Major Groove Binding* has a path of seven IS-A links to the root concept *Biological Proces*s of the top area. The concepts along the path accumulate more complexity in their nature and definition as we get farther away from the root. From a linguistic or logical perspective, one could characterize the additional complexity as expanding intension [not intention] as we move down the hierarchy. In this light, we hypothesize that the likelihood of a missing relationship error increases with the additional complexity associated with the increasing distance from the root. In other words, one can expect a higher percentage of concepts with missing relationships when going down the path.

To formalize the above idea, we define the "level" of a concept as the number of IS-A links in the path from the concept to its root. Thus, in Fig. 5, the levels of *DNA Binding* and of *DNA Major Groove Binding* are five and seven, respectively. By definition, the root, *Biological Process*, resides at Level 0. (When a concept has multiple parents—and hence there are multiple paths to the root—its longest path defines its level. Topological sort [33] can be used to calculate the longest-path distance for all concepts in the top area in linear time.) It follows that a concept with a higher level number appears lower in the diagram of its path to the root.

To make a binary distinction between more complex and less complex concepts, we divide the levels of the hierarchy into two halves, the higher-indexed and lower-indexed halves, with the expectation of more missing relationships in the higher-indexed-half of the hierarchy where concepts are more complex (and *lower in the diagram*). This provides us with a practical tool for QA in cases where the top area is too large to be reviewed in its entirety.

In a top area with long concepts paths it is recommended that QA processing be concentrated on the higher-indexed levels, since their concepts are more complex and are expected to have more missing relationships. We formulate this as Hypothesis 2. We start with two definitions.

The phrase “higher-indexed-half levels” refers to the levels $\left\lfloor {\frac{n + 1}{2}} \right\rfloor$, $\left\lfloor {\frac{n + 1}{2}} \right\rfloor$ + 1, …, *n*, whereby there are *n* levels in total, including Level 0 of the root, in the longest path in the top area. These are the levels far from the root.

The expression “lower-indexed-half levels” describes the levels 0, 1, …, $\left\lfloor {\frac{n + 1}{2}} \right\rfloor$ − 1. These levels are closer to the root. We arbitrarily chose to round down, which is not problematic as long as it is done consistently in both definitions.

For example, there are 10 levels in the top area of the NCIt BP hierarchy. The lower-indexed-half levels are 0, 1, 2, 3, and 4 and the higher-indexed-half levels are 5, 6, 7, 8 and 9. For the top area of the *Eye/vision finding* subhierarchy, there are 11 levels. The lower-indexed-half levels are Levels 0 to 5 and the higher-indexed-half levels are Levels 6 to 10.

#### Hypothesis 2

Concepts in the higher-indexed-half levels of the top area have a higher likelihood of missing relationship errors than concepts in the lower-indexed-half levels.

In the study of the NCIt’s BP hierarchy, we used the complete top area of 513 concepts as our first testbed to evaluate Hypothesis 2. All concepts of its top area were reviewed for missing relationships. We determined the numbers of erroneous concepts found in each level and their percentages. Similarly, we also performed the statistical analysis on the random sample of 96 concepts from the top area of the SNOMED CT’s *Eye/vision finding* subhierarchy to test Hypothesis 2.

The method of QA implied by Hypotheses 1 and 2 is powerful, because its beneficial effect goes beyond the actually considered concepts in the top area. If it is determined that a concept *C* from the top area is missing a relationship *R* pointing to a target *D*, then all of *C*’s descendant concepts *inside and outside of the top area*, should also have the relationship *R*, and if they do not have it, these are cases of missing relationship errors. When fixing these errors, the relationship *R* will either point to the same target *D* or to a descendant of *D*.

All the descendants of *C* can be identified algorithmically and presented to the ontology curator to approve the addition of *R* to them. Unless there is another error in the IS-A hierarchy itself, this approval should be granted in every case, making the process easy for the curator. We will demonstrate this effect in the Results for the NCIt *Biological Process* hierarchy.

## Results

### Top area concepts and control sample in the NCIt’s *Biological Process* hierarchy

The results for the *Biological Process* hierarchy of NCIt are summarized in Table 2, which shows the level distribution of concepts in the top area and the number of concepts found to be missing relationships at the different levels. For example, at Level 5, consisting of 88 concepts, we found 61 (69.3%) that were missing relationships. Out of the 513 concepts in the top area, 45.2% were found to be missing relationships.

**Table 2:** Missing relationship error distribution by level in the top area of NCIt’s BP hierarchy

Level	# concepts	# concepts missing relationships	% of concepts missing relationships
0	1	0	0
1	7	0	0
2	69	15	21.7
3	138	53	38.4
4	125	58	46.4
5	88	61	69.3
6	44	32	72.7
7	14	8	57.1
8	23	5	21.7
9	4	0	0
Total	513	232	45.2

At levels 0 and 1 there are very general concepts that "rightfully" have no relationships. For example, two such concepts at Level 1 are *Regulatory Process* and *Pathologic Process*. For levels 2 to 6 the percentages of concepts with missing relationship errors increases monotonically. At levels 7, 8, and 9, this reverses, presumably due to the low absolute numbers of concepts.

Table 3 lists the numbers of concepts reported as having missing relationship errors for each different kind of relationship according to (YC), and how many of them were confirmed by the secondary expert reviewer (SdC). For example, 103 concepts were deemed to be missing the relationship *Location*, but only 84 of these were confirmed in the secondary review. The largest numbers of missing relationships in the initial QA analysis were *Location* (missing 103 times) and *Part of Process* (missing 113 times). (SdC) agreed only with 82% of the missing *Location* relationships and only with 50% of the missing *Resulting Chemical or Drug* relationships. However, we recently checked the most recent NCIt release (20.06e) and found that 129 top-area concepts in the 15.02d release have now been added the relationship *Part of Process* with the target *Biochemical Process* inspired by our study.

**Table 3:** Number of concepts in the NCIt’s BP top area reported missing relationship for each relationship type

Relationship	# concepts missing relationship	# concepts confirmed by (SdC)
*Location*	103	84
*Initiator Chemical or Drug*	1	0
*Initiator BP*	2	0
*Resulting Anatomy*	1	1
*Resulting BP*	3	1
*Resulting Chemical or Drug*	20	10
*Part of Process*	113	4
Total	232	99

In Table 4, there are examples of concepts that are missing relationships, as confirmed in the secondary review of (SdC). For example, *ABC Transporter Binding* should have the relationship *Part of Process* to *Biochemical Process*.

**Table 4:** Examples of concepts confirmed to have missing relationships in the NCIt’s BP top area for different relationships by (SdC)

Relationship	Example confirmed concept missing relationship	Target of missing relationship
*Location*	*Adrenal Hormone Activity Induction*	*Adrenal Gland*
*Resulting Anatomy*	*Coagulation Process*	*Fibrin*
*Resulting Chemical or Drug*	*Histamine Production*	*Histamine*
*Part of Process*	*ABC Transporter Binding*	*Biochemical Process*

Table 5 shows counterexamples for which (SdC) provided reasons why relationships should not be added. Thus *Glucocorticoid Secretion Process* is not missing the *Resulting Chemical or Drug* relationship (directed to *Glucocorticoid*). The reason is as follows. In order for a product (e.g., a hormone) to be secreted, it first has to be produced. However, the set of processes (and enzymes) involved in production may be different from those involved in secretion. (Thyroid hormone is a good example of a product where production and secretion are two completely separate processes.)

**Table 5:** Rejected examples of concepts missing relationships in the NCIt’s BP top area for different relationships by (SdC)

Relationship	Reported example of concept missing relationship	Proposed target of missing relationship	Reason
*Location*	*RNA Processing*	*Nucleus*	Not always true
*Resulting BP*	*Antigen Binding*	*Immune Response Process*	Not always true
*Resulting Chemical or Drug*	*Glucocorticoid Secretion Process*	*Glucocorticoid*	Secretion processes do not produce chemicals
*Part of Process*	*Defecation*	*Gastrointestinal Process*	*Gastrointestinal Process* is the parent of *Defecation*

Making decisions about modeling errors requires complex human thought processes. Thus, different experts can come to different plausible conclusions. For example, in the last row of Table 5, *Defecation* can be viewed as a child of *Gastrointestinal Process,* but it can also be modeled as a *Part of Process* of the comprehensive concept *Gastrointestinal Process.* The decision of (SdC), follows precedents established during the overall conceptualization of the *Biological Process* hierarchy.

Only 13 of the 100 control concepts were determined to be missing relationships. Table 6 is a contingency table for the control concepts, which are not from the top area, and the study concepts. With Fisher’s exact two-tailed test [34] we computed a *p*-value < 0.0001, establishing statistical significance. In other words, the concepts in the top area are significantly more likely to have missing relationship errors than concepts in the other sampled areas. Thus, Hypothesis 1 is confirmed.

**Table 6:** The 2 × 2 contingency table for the concept errors in NCIt’s *Biological Process* top area versus concepts from other areas of the area taxonomy

	# erroneous concepts	# concepts w/o errors
Non-top areas	13	87
Top area	232	281

Advancing to Hypothesis 2, Table 7 summarizes the comparison between concepts at levels 0 to 4 missing relationships versus concepts at levels 5 to 9 missing relationships. There are 340 concepts in levels 0 to 4, which is nearly twice as many as concepts in the levels 5 to 9. However, the percentage of concepts in levels 5 to 9 missing relationships (61.3%) is higher than that in levels 0 to 4 (37.1%), confirming Hypothesis 2. To establish statistical significance, we used the same approach as for Hypothesis 1 and computed a *p*-value < 0.0001 by Fisher’s test. Thus, the results confirm Hypothesis 2 that concepts in the higher-indexed-half levels of the top area have a significantly higher likelihood of missing relationships than those in the lower-indexed-half levels.

**Table 7:** The 2 × 2 contingency table for concept errors between the lower-indexed-half levels and higher-indexed-half levels

Level range	# erroneous concepts	# concepts w/o errors	Error percentage
0–4 (lower-indexed-half)	126	214	37.1
5–9 (higher-indexed-half)	106	67	61.3

### QA study on the SNOMED CT’s *Eye/vision finding* subhierarchy

After the two-step review on the random sample of 96 top area concepts and 96 concepts outside the top area, we found that there were 42 top area concepts (43.75%) and 24 non-top area concepts (25%) missing relationships. The two-tailed *p*-value of Fisher’s exact test is 0.0095. Hence, Hypothesis 1 was also confirmed for the SNOMED CT’s *Eye/vision finding* subhierarchy, i.e., the top area concepts are significantly more likely to have missing relationship errors than concepts in other areas.

Table 8 summarizes the distribution of all top area concepts, of the audited concepts, and of the erroneous concepts among them in terms of the level. The 1301 top area concepts are distributed over 11 levels, including the root concept *Eye/vision finding* at Level 0. For example, there are 323 concepts at Level 5, i.e., having a path of five IS-A relationships to the root concept, out of which 29 (8.98%) were randomly selected for auditing. Our domain experts found that eight of them (27.59%) did miss relationships.

**Table 8:** The QA study results on the SNOMED CT’s *Eye/vision finding* subhierarchy

Level	# concepts	# audited concepts	% of concepts audited	# concepts missing relationships	% of concepts missing relationships
0	1	0	0	0	
1	19	0	0	0	
2	58	0	0	0	
3	132	8	6.06	6	75
4	250	18	7.20	6	33.33
5	323	29	8.98	8	27.59
6	272	19	6.99	9	47.37
7	165	18	10.91	11	61.11
8	54	4	7.41	2	50
9	25	0	0	0	
10	2	0	0	0	
Total	1301	96	7.38	42	43.75

According to Table 8, there are 20 concepts missing relationships out of 55 audited concepts (36.36%) in the levels 0 to 5, and 22 erroneous concepts out of 41 audited concepts (53.66%) in the levels 6 to 10. Although the two-tailed *p*-value of Fisher’s exact test is greater than 0.05, the error rate of the higher-indexed-half levels is almost 1.5 times the error rate of the lower-indexed-half levels.

Table 9 lists five example concepts in the *Eye/vision finding* top area, each of which was reported missing two relationship types. For example, the concept *Enophthalmos due to orbital tissue atrophy* at Level 5 in the top area was reported missing the relationship *Due to* pointing to *Atrophy of soft tissue of orbit* and the relationship *Associated morphology* pointing to *Posterior displacement*. Although we did not report our finding of errors to SNOMED CT, checking the most current release January 2020 International Edition, we found that 23 out of 42 erroneous concepts identified in our study have been corrected, confirming our study domain experts’ suggestions, including all the five examples in Table 9.

**Table 9:** Five example concepts in the *Eye/vision finding* top area missing two relationships

Concept	Level in the top area	Missing relationship type 1	Target 1	Missing relationship type 2	Target 2
Normal intraocular pressure	3	Interprets	Intraocular pressure	Has interpretation	Normal
Decreased red reflex	3	Interprets	Red reflex	Has interpretation	Decreased
Irregular tear film	4	Interprets	Ocular tear film observable	Has interpretation	Abnormal
Enophthalmos due to orbital tissue atrophy	5	Due to	Atrophy of soft tissue of orbit	Associated morphology	Posterior displacement
Impairment level: better eye: severe impairment: lesser eye: total impairment	7	Interprets	Visual function	Has interpretation	Impaired

### Further QA opportunities after discovering concepts missing relationships in the top area

In the NCIt *Biological Process* hierarchy, 354 of 513 top area concepts (69%) are leaves, i.e., they have no IS-A children. Thus, adding relationships to them would affect only them. However, there are 68 concepts among the remaining 159 non-leaf concepts that were missing relationships, which affects their children and descendants (if they exist) also. It is, however, possible that children and descendants already have the correct relationships.

The results of investigating this question are shown in Table 10. Five of the 68 concepts have descendants only in the non-top areas (line 1). Another 40 concepts (line 3), have all their descendants in the top area. The remaining 23 concepts (line 2) have some descendants in the top area and others outside. The number of affected descendants in the last column (Table 10) is the sum of the descendant concepts missing the same relationships as their ancestors and the number of descendants having the relationships, but with incorrect targets. Incorrect targets are different from their ancestor's targets, but not more specific than them.

**Table 10:** Affected descendants of the 68 non-leaf concepts missing relationships in the NCIt’s BP top area

	# concepts	Total # descendants outside top area	# affected descendants
All descendants are in non-top areas	5	15	5
Some descendants are in top area	23	102	50
All descendants are in the top area	40	N/A	N/A
Total	68	117	55

## Discussion

### Applicability of QA with large top areas

In the NCIt there are 11 hierarchies for which lateral relationships are defined (Table 11). For SNOMED CT, there are eight such hierarchies (Table 12). Both tables show the numbers and percentages of concepts in the top areas for their area taxonomies. For example, NCIt’s *Conceptual Entity* hierarchy has 12,409 concepts, of which 8851 (71.3%) are in the top area. In SNOMED CT, for example, the *Clinical finding* hierarchy contains 114,397 concepts, of which only 6427 (5.6%) are located in its top area.

**Table 11:** Top areas of 11 hierarchies in NCIt (15.02d release)

Hierarchy	# concepts	# concepts in top area	%
*Activity*	10,633	10,087	94.9
*Anatomic Structure, System, or Substance*	6747	1730	25.6
*Biological Process*	1145	513	44.8
*Chemotherapy Regimen or Agent Combination*	3419	41	1.2
*Conceptual Entity*	12,409	8851	71.3
*Disease, Disorder or Finding*	25,360	14,347	56.6
*Drug, Food, Chemical or Biomedical Material*	17,681	16,139	91.3
*Experimental Organism Diagnosis*	1701	327	19.2
*Gene*	8914	395	4.4
*Gene Product*	5256	90	1.7
*Molecular Abnormality*	1244	192	15.4

**Table 12:** Top areas of eight hierarchies in SNOMED CT (2020-01-31 release)

Hierarchy	# concepts	# Concepts in top area	%
*Body structure*	39,323	27,224	69.2
*Clinical finding*	114,397	6427	5.6
*Event*	3189	3006	94.3
*Observable entity*	9144	8744	95.6
*Pharmaceutical / biologic product*	22,244	418	1.9
*Procedure*	58,154	2628	4.7
*Situation with explicit context*	4739	61	1.3
*Specimen*	1702	34	2.0

In the NCIt, all hierarchies except for the *Chemotherapy Regimen or Agent Combination* hierarchy (1.2% in top area) and the *Gene Product* hierarchy (1.7%) have disproportionally large top areas. In SNOMED CT this anomaly also exists, with the exception of the *Situation with explicit context* hierarchy (1.3% in top area) and the *Specimen* hierarchy (2.0%). Hence, the described characteristic is applicable for QA of nine NCIt and six SNOMED CT hierarchies. Given that according to Elhanan et al. [35] missing SNOMED CT relationships were considered detrimental in a user study, QA of those hierarchies is recommended.

Terminologies such as the NCIt are driven by the needs of its users, as opposed to abstract modeling criteria. Thus, concepts that are requested by users are included, even if they are not fully defined relative to existing concepts. In description logic parlance they are primitive concepts that are therefore "under-modeled." Among the 96 SNOMED CT top area concepts, 16 are fully defined, of which seven (43.75%) were found missing relationships, and out of the other 80 primitive concepts, 35 (43.75%) were reported missing relationships. For the 96 non-top area concepts, the respective numbers of fully defined concepts and primitive concepts are 42, of which four concepts, i.e., 9.52% were missing relationships, and 54, of which 20 concepts, i.e., 37.04% were missing relationships). As mentioned in [36], the abstraction networks do not differentiate primitive concepts from fully defined concepts.

Even an under-modeled concept without well-specified relationships is very useful as a "hook" on which to hang preferred terms, synonyms, definitions, and parent/child relationships. However, *in extremis* such concepts are not assigned any relationships and therefore will end up in the top area of the area taxonomy. This can be seen for NCIt’s *Activity* and *Drug, Food, Chemical or Biomedical Material* hierarchies (Table 11). We also see such cases in SNOMED CT: the *Event* and *Observable entity* hierarchies (Table 12). In such cases, we deem the hierarchies not to warrant QA processing via our approach. Clearly, conscious decisions have been made by the curators to leave these hierarchies almost entirely primitive.

### Error correction by inheritance

Returning to Table 10, we quantified the missing relationship errors due to inheritance from the top area to other areas. There are 232 concepts that are missing relationships. Of those 164 (70.7%) are leaves. Leaves cannot cause inheritance of missing relationships into other lower areas. However, these concepts, will move to other areas of the area taxonomy when they are given the proper sets of relationships.

The 68 non-leaf concepts have 117 descendants in other areas. All of the descendants *could be* targets of inheritance of relationships added to the 68 concepts in the process of correcting them. However, in some cases the descendant concepts *already have* those relationships. In other words, modeling errors made for concepts in the top area are not always repeated at lower levels. Only for 55, out of 117, descendant concepts are relationships missing, and these errors are automatically corrected by inheriting the missing relationships to them.

Had the missing relationships been defined by the editors at the highest possible positions in the hierarchy, then the work of adding them to the 117–55 = 62 other concepts would have been saved, which would have been automatically inferred by the classifier.

Thus, the impact of the inheritance of the missing relationships is much higher than it appears to be when looking at Table 10. The question remains whether those missing relationships, had they been assigned at a higher level, would have applied to all the children. This question must be left to future research.

An interesting question raised by an anonymous reviewer is out of those concepts reported missing relationships, how many missed the same lateral relationships as their ancestors which were also identified missing relationships. For the SNOMED CT study, those erroneous concepts by chance have no hierarchical relationships. This is possible since the number of reviewed top area concepts (96) is only 7.38% of all top area concepts and only 2.49% of non-top area concepts (96) were reviewed. While for the NCIt study, out of the 13 erroneous non-top area concepts, only one was identified missing the same lateral relationship as its ancestor in the top area. Six concepts were reported missing the same kind of lateral relationship as their ancestors but with more specific targets. The remaining six concepts’ ancestors had no error. Out of the 232 erroneous top area concepts, 88 were reported the same error as their ancestors, 23 missed the same kind of relationship as their ancestors but with more specific targets, and 30 were reported missing additional relationships in addition to those for their ancestors.

### Impact of error correction on the area taxonomy

**Fig. 6:**
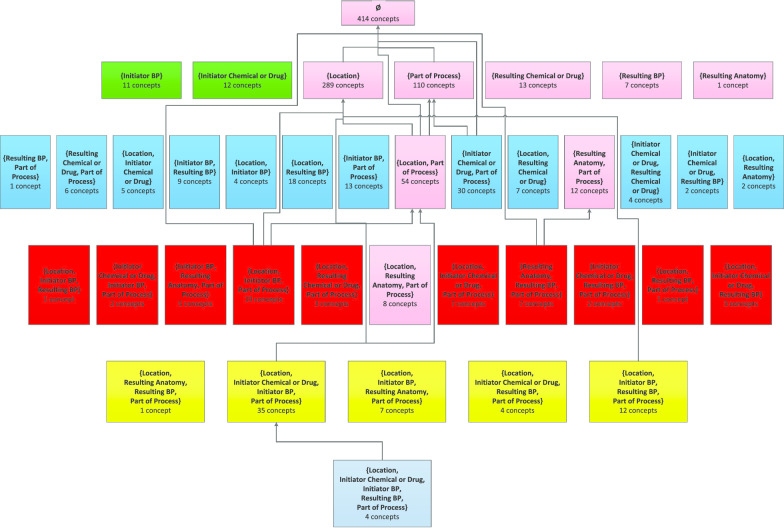
Revised area taxonomy for the NCIt BP hierarchy incorporating the confirmed corrections. Pink highlights the areas that are different from the original in Fig. 3

In this paper, in contrast to [23], we chose to use the top area of the taxonomy as the characterization for the set of all concepts not having any relationships, and accordingly we framed the anomaly as that of having a large top area. This description provides better context to the research. For example, it enables us to use the area taxonomy of the *Biological Process* hierarchy (Fig. 6) to illustrate the changes that occurred as a result of our QA analysis, including corrections in the non-top areas due to the inheritance of the additional relationships. We note that the taxonomy abstraction networks do not themselves provide inherent QA methodologies; they just enable the identification of sets of concepts that are highly likely to have more errors than the rest of the hierarchy. Examples of other such sets include small partial-areas [21] and overlapping concepts [22], both described in our taxonomy framework.

The review of (SdC) confirmed the missing relationships for 99 concepts (42.7% = 99/232) in the top area and 10 concepts (76.9% = 10/13) in the non-top areas (see Tables 6 and 13). Although only a portion of the missing relationship errors found in our analysis for both the top area and the non-top (control) areas were confirmed by (SdC), their number (Table 13) is still sufficient for statistical significance. The two-tailed *p*-value = 0.0311 by Fisher’s test is, however, much higher than that derived for Table 6.

**Table 13:** The 2 × 2 contingency table for erroneous concepts in the top area and non-top areas confirmed by (SdC)

	# erroneous concepts	# concepts w/o errors	Total concepts in the study
Non-top areas	10	90	100
Top area	99	414	513

Figure 6 shows the revised area taxonomy based on the version of the NCIt after the confirmed corrections have been implemented locally at our site. All areas where concepts have changed are highlighted in pink. Those changes reflect both the concepts that have moved out of the top area and the concepts that have inherited new relationships and thus have moved from one non-top area to another on Levels 2 and 3 (pink areas). Of special note are the new Level 1 area {*Resulting Anatomy*} that did not exist in Fig. 3 and the increase in the size of the area {*Location*} from 207 to 289 concepts.

### Internal versus external review

The relatively high degree of disagreement between the primary expert reviews and secondary expert reviews requires an explanation. The external reviewer who did the primary review (YC) has no information about the ontology design, except for the ontology itself. The result of the external reviewer also has no impact on the future workload of the ontology team. Thus, the external reviewer is unencumbered and can freely report any modeling details for which there is a possibility of an internal inconsistency or an incongruence with the real world.

The secondary review was done by the main internal NCIt expert (SdC) who has a considerable amount of knowledge on the design of the ontology that goes beyond the ontology itself. This knowledge may include experience reports of previous maintenance regimens, style preferences of the staff members, and informal guidelines. The needs and past requests of the users of the ontology are also known to the internal expert, but not to the external expert. Thus, when reviewing the external error report, the (internal) curator takes all these additional sources of knowledge into account.

For example, the main topic of the NCIt is "cancer," and therefore the depth of coverage of non-neoplasm concepts in some hierarchies is limited relative to the many neoplasm concepts in the *Disease, Disorder or Finding* hierarchy. Moreover, the NCIt curators do not necessarily add an ontological element, even if it is a correct assertion, unless it is needed for a logical definition or reasoning or required for a use-case. In some situations, relationships could be added, but they might not add much meaning for a targeted end user and would take more effort to maintain later on.

In summary, it is not unexpected that only a portion of the externally reported errors were accepted by (SdC) in the current study. This explains why the *p*-value obtained for the confirmed errors is much higher than the *p-*value for the errors reported by the external domain expert.

### Improving the efficiency of the QA review

Our domain experts found the QA work to be quite time consuming. As an enhancement to our approach, it would be good to add an automated component to narrow down the search space by suggesting concepts that warrant attention—and thus make the review faster. Hypothesis 2 points towards a method for reducing the effort. Curator should concentrate on reviewing the higher-indexed-half levels of the top area when there is a very large top area. Such a methodology is expected to yield a higher ratio of errors than when reviewing a random set of top area concepts of the same size.

For the QA study on the SNOMED CT *Eye/vision finding* subhierarchy, the *p* value for Hypothesis 2 is slightly higher than 0.05, although the error rate of the higher-indexed-half levels is much higher than that of the lower-indexed-half levels. One possible reason is that the sample of 96 concepts is too small. Having the same percentages of erroneous concepts for a sample of double the size, would have shown statistical significance.

The bottom-most levels in the top area should be especially prone to missing relationship errors. Thus, we asked the reviewers to audit all the concepts in those two levels. They reviewed all 25 concepts at Level 9 and all two concepts at Level 10. The result was that 17 concepts at Level 9 (68%) and all two concepts at Level 10 (100%) were found to be missing relationships. These percentages added anecdotal evidence that the higher-indexed-half levels tend to have more errors than the lower-indexed-half levels, supporting Hypothesis 2.

### Future research

Zhe et al. [37] and Ochs et al. [38] presented the meta-ontology of families for the ontologies hosted in the BioPortal [20] ontology repository. They have demonstrated the scalability of a specific QA technique to a whole family of such ontologies, by showing that it was successful for six out of six ontologies of that family. The technique in this paper was shown to be successful for one hierarchy and one subhierarchy of two ontologies. Therefore this technique should be tested for at least four more ontologies, to attempt to demonstrate scalability to a whole family of ontologies.

## Conclusions

Quality assurance (QA) is an important step in an ontology’s life cycle. Due to the complexity and the large size of many ontologies, automated and semi-automated tools for supporting ontology QA are essential. In this paper, we focused on auditing one single kind of omission error: missing relationships. The foundation of our approach was an abstraction network called an area taxonomy and its variation called a subtaxonomy. An anomalous feature in an area taxonomy (or a subtaxonomy), a large top area, was used as an indicator for guiding the search for missing relationships. The methodology was demonstrated for the NCIt’s *Biological Process* hierarchy and the SNOMED CT *Eye/vision finding* subhierarchy. A statistically significantly larger number of missing relationship errors in the top area than for a control sample was identified in both studies. This methodology can be seen as a useful addition to the arsenal of tools available to QA personnel.

## Data Availability

The datasets used and/or analysed during the current study are available from the corresponding author on reasonable request.

## Abbreviations

QA: Quality Assurance
NCIt: National Cancer Institute thesaurus
SNOMED CT: SNOMED Clinical Terms
BP: Biological Process hierarchy of National Cancer Institute thesaurus
